# Can Immobilized Artificial Membrane Chromatography Support the Characterization of Antimicrobial Peptide Origin Derivatives?

**DOI:** 10.3390/antibiotics10101237

**Published:** 2021-10-12

**Authors:** Krzesimir Ciura, Natalia Ptaszyńska, Hanna Kapica, Monika Pastewska, Anna Łęgowska, Krzysztof Rolka, Wojciech Kamysz, Wiesław Sawicki, Katarzyna E. Greber

**Affiliations:** 1 Department of Physical Chemistry, Medical University of Gdańsk, Al. Gen. Hallera 107, 80-416 Gdańsk, Poland; hanna.kapica@gumed.edu.pl (H.K.); monika-pastewska@gumed.edu.pl (M.P.); wsawicki@gumed.edu.pl (W.S.); 2 QSAR Lab Ltd., Trzy Lipy 3 St., 80-172 Gdańsk, Poland; 3 Department of Molecular Biochemistry, Faculty of Chemistry, University of Gdansk, Al. Gen. Hallera 107, 80-308 Gdańsk, Poland; natalia.ptaszynska@ug.edu.pl (N.P.); anna.legowska@ug.edu.pl (A.Ł.); krzysztof.rolka@ug.edu.pl (K.R.); 4 Department of Inorganic Chemistry, Medical University of Gdańsk, Al. Gen. Hallera 107, 80-416 Gdańsk, Poland; Wojciech.kamysz@gumed.edu.pl

**Keywords:** IAM-HPLC, antimicrobial peptide, cell-penetrating peptide (CPP), transportan 10 (TP10-NH2), short cationic lipopeptides

## Abstract

The emergence and spread of multiple drug-resistant bacteria strains caused the development of new antibiotics to be one of the most important challenges of medicinal chemistry. Despite many efforts, the commercial availability of peptide-based antimicrobials is still limited. The presented study aims to explain that immobilized artificial membrane chromatography can support the characterization of antimicrobial peptides. Consequently, the chromatographic experiments of three groups of related peptide substances: (i) short cationic lipopeptides, (ii) citropin analogs, and (iii) conjugates of ciprofloxacin and levofloxacin, with a cell-penetrating peptide were discussed. In light of the discussion of the mechanisms of action of these compounds, the obtained results were interpreted.

## 1. Introduction

The development of new antimicrobial agents is one of the most critical challenges of medicinal chemistry. Several natural and synthetic compounds have been explored and investigated to find new, effective, and safe antimicrobial agents. The antimicrobial peptides, lipopeptides, and other peptide origin derivatives, such as peptidomimetics, are very promising structures among the tested substances. They showed severe therapeutic potential due to their broad spectrum of activity, rapid bacterial killing, and synergy with classical antibiotics [1,2,3]. Generally, the antibacterial mechanism of action of peptides and lipopeptides is mainly connected with the interactions between peptides and bacterial membranes [4], but some studies showed different results [5]. Nonetheless, the most recognized mechanisms of action are the barrel-stave model, carpet model, and toroidal model for killing pathogenic bacteria organisms [6].

Understanding the physicochemical and structural properties of peptides is an essential requisite for the rational design of active derivatives. Nevertheless, their lipophilicity is challenging to analyze using traditional in silico or octanol/water partition coefficients. Consequently, the estimation of their in vivo distribution and permeability is also difficult [7]. Except for family cell-penetrating peptides (CPP) [8], their peptide derivatives also often showed limited cell permeability due to their polar nature. For this reason, the assessment of phospholipid’s affinity for peptide derivatives is critical. Generally, the partitioning systems containing phospholipids as the organic phase, like liposomes or cell culture techniques, can mimic the interactions between peptides and biological membranes [9].

Nevertheless, nowadays, the most popular approach involves immobilized artificial membrane HPLC (IAM-HPLC). Several advantages of IAM-HPLC include complete automation, a short analysis time, and excellent lab-to-lab reproducibility. What is more, Valko and coworkers reported promising results of an phospholipid-binding study of potential peptide therapeutics using IAM-HPLC [10,11,12,13,14,15,16].

This study continues our research program focused on assessing the physicochemical properties of drug candidates using chromatographic and biochromatographic approaches [17,18,19,20,21,22,23,24]. In this study, we investigated the possibility of using IAM-HPLC for the characterization of three groups of related peptide substances: (i) short cationic lipopeptides, (ii) citropin analogs, and (iii) conjugates of ciprofloxacin (CIP) and levofloxacin (LVX) with a cell-penetrating peptide named transoprtan 10 (TP10-NH_2_) and its analog extended at *N*-terminus by l-cysteine synthesized in our and collaborated laboratories.

## 2. Results

In Table 1, the experimentally determined time of retention in the investigated IAM-HPLC system and calculated using calibration cure chromatographic hydrophobicity indices of IAM (CHI_IAM_) was noticed. In short, cationic lipopeptides have strong interactions between the stationary phase and analytes occurring and do not migrate. The conjugates of ciprofloxacin and levofloxacin with a cell-penetrating peptide behaved completely contarry. All target derivatives (six conjugates of CIP and LVX with TP10-NH_2_) migrated to the front of the mobile phase. The retention time of the citropin analogs ranged from 3.349 to 4.495 min, which referred to 27.85–45.95 CHI_IAM_, respectively. 

## 3. Discussion

Several pharmaceutical and biotechnological companies are currently looking for new modalities outside the traditional small molecular drug space [7]. However, the obtained results seem to look disappointing, as typical negative experiments since the quantitative data were obtained for only one series of the tested compounds. It could be beneficial to understand the possible mechanisms of action better. In the case of short cationic lipopeptides, there are probably occurring simultaneous hydrophobic interactions between the hydrocarbon chains and interactions between the positively charged amino acid head and the negatively charged phosphate group present in the structure of phosphatidylcholine. The combination of these interactions causes very strong interactions with the phospholipids. Interestingly, both active and nonactive substances can not be eluted from IAM only using the 100% organic phase [5,25]. This finding suggested that the mechanism of action of short cationic lipopeptides is not trivial and meets our earlier results as to its more complex nature than just the simple surfactant [5,25]. An interesting situation was observed in the case of the conjugates of CIP and LVX with transportan 10. The parent fluoroquinolone antibiotics showed a moderate affinity to the phospholipids: 24.88 and 19.70 CHI_IAM_ for LVX and CIP. However, when covalently linked with TP10-NH_2_, they lost their affinity to the phospholipids. At the same time, the synthesized conjugates remained highly biologically active [26]. It is worth highlighting that fluoroquinolone antibiotics may also penetrate bacterial cells in a hydrophilic way using porin channels [27,28]. The latter can explain the visible activity of the tested conjugates. This assumption is supported by the observation of improved dissolution in the water of the designed conjugates compared to the started fluoroquinolone antibiotics structures. Among the tested substances, citropin analogs characterized a moderate to relatively high affinity to stationary IAM. These results indicated that the citropin derivatives should penetrate the biological membranes. 

## 4. Materials and Methods

### 4.1. Materials and Analytes

#### 4.1.1. Short Cationic Lipopeptides

The lipopeptides sequences were de novo designed and synthesized using the 9-fluorenylmetoxycarbonyl (Fmoc) methodology on the Fmoc-Rink Amide AM resin (0.59 mmol/g, IrisBiotech, Marktredwitz, Germany) [29]. To remove the Fmoc group from the protected amino acid residue, a 20% solution of piperidine in *N*,*N*-dimethyloformamide (DMF) was used. A peptide bond was created by in situ activation with the diizopropylocarbodoimide/1-hydroxybenzotriazole (DIC/HOBT) procedure. Deanchoring of the lipopeptides from the solid support and deprotection of the amino acid side chains were achiewed by treating the lipopeptidyl resin with the mixture of trifluoroacethic acid (TFA; 95%), triizopropylosilane (TIS; 2.5%), and water (2.5%) for 1 h. The deanchoring mixture was then drained, concentrated on a rotary evaporator (Heidolph, Schwabach, Germany), and treated with cold diethyl ether to precipitate the lipopeptides. The precipitated lipopeptides were dissolved in water and freeze-dried (Christ, Martinsried, Germany). 

Purification of the synthesized lipopeptides was carried out by semipreparative reverse-phase high-performance liquid chromatography (RP-HPLC) on a C8e column (Macharey-Nagel, Düren, Germany) with a linear gradient (20–60%) of acetonitrile in water (both solvents contained 0.1% TFA). The identity of the obtained lipopeptides was confirmed via mas spectrometry (MALDI-TOF, Bruker Daltonics, Ettlingen, Germany). The sequences of the target antimicrobial lipopeptides are presented in Table 2.

#### 4.1.2. Citropin Analogs

The citropin analogs were assembled manually by solid-phase procedures on a polystyrene AM-RAM resin (0.66 mmol/g, Rapp Polymere, Tuebingen, Germany) using 9-fluorenylmetoxycarbonyl (Fmoc) methodology [29].

The Fmoc group was removed from the protected amino acid residue by a 20% solution of piperidine in *N*,*N*-dimethyloformamide (DMF). A peptide bond was created by the in situ activation with the 2-(1*H*-benzotriazol-1-yl)-1,1,3,3-tetramethyluronium hexafluorophosphate/*N*,*N*-diisopropylethylamine (HBTU/DIPEA) procedure. Deanchoring of the peptides from the solid support and deprotection of the amino acid side chains were achieved by treating the peptidyl resin with the mixture of trifluoroacethic acid (TFA; 95%), triizopropylosilane (TIS; 2.5%), and water (2.5%) for 2 h. The deanchoring mixture was then drained, concentrated on a rotary evaporator (Heidolph, Schwabach, Germany), and treated with cold diethyl ether to precipitate the peptides. The precipitated peptides were dissolved in 2% ACN and freeze-dried (Christ, Martinsried, Germany).

Purification of the synthesized peptides was carried out by semipreparative reverse-phase high-performance liquid chromatography (RP-HPLC) on a C18e column (Macharey-Nagel, Düren, Germany) with a linear gradient (20–40%) of acetonitrile in water (both solvents contained 0.1% TFA). Each citropin analog was analyzed by RP-HPLC and matrix-assisted laser-desorption ionization time-of-flight mass spectrometry MALDI-TOF (Bruker Daltonics, Ettlingen, Germany). The sequences of the investigated citropin analogs are presented in Table 3.

#### 4.1.3. Conjugates of Ciprofloxacin (CIP) and Levofloxacin (LVX) with a Cell-Penetrating Peptide

TP10-NH_2_ and CTP10-NH_2_ were obtained using the standard Fmoc chemistry solid-phase peptide synthesis (SPPS) utilizing an automatic prelude peptide synthesizer ((Gyros) Protein Technology Inc., Tucson, AZ, USA) and have been described previously [26]. The peptides were synthesized on a TentaGel S RAM resin (substitution 0.24 meq/g, Rapp Polymere, Germany) to obtain peptides with an amide group on the C-terminus after cleavage. After completing the synthesis, the peptides were removed from the resin in a one-step procedure using a mixture of TFA:phenol:triisopropylosilane:H_2_O (88:5:2:5, *v/v/v/v*). The obtained peptides were purified using PLC 2050 Gilson HPLC with Gilson Glider Prep. software (Gilson, Villiers le bel, France). The device was provided with a Grace Vydac C18 (218TP) HPLC column (22 mm × 250 mm, 10 µm, 300 Å, Resolution Systems). The solvent systems were 0.1% TFA in water (A) and 80% acetonitrile in A (B). Different linear gradients were applied (flow rate 20 mL min^−1^ monitored at 226 nm). The homogeneity of the compounds was examined with the HPLC Pro Star system (Varian, Mulgrave, Australia) and using a Kinetex 5-μm XB-C18 100 Å column (4.6 mm × 150 mm, Phenomenex^®®^, Torrance, CA, USA). A linear gradient from 10 to 90% B for 40 min with a flow rate of 1 mL min^−1^ monitored at 226 nm was utilized. The synthesized peptides had a purity of at least 95%. The correctness of the molecular masses of the synthesized compounds was confirmed by a mass spectrometry analysis using MALDI-TOF MS (Biflex III MALDI-TOF spectrometer, Bruker Daltonics, Ettlingen, Germany or MALDI TOF/TOF 5800+ spectrometer, AB SCIEX, Framingham, Massachusetts, USA), with an α-cyano-4-hydroxycinnamic acid (CCA) and/or 2,5-dihydroxybenzoic acid (DHB) matrix.

**Figure 1:**
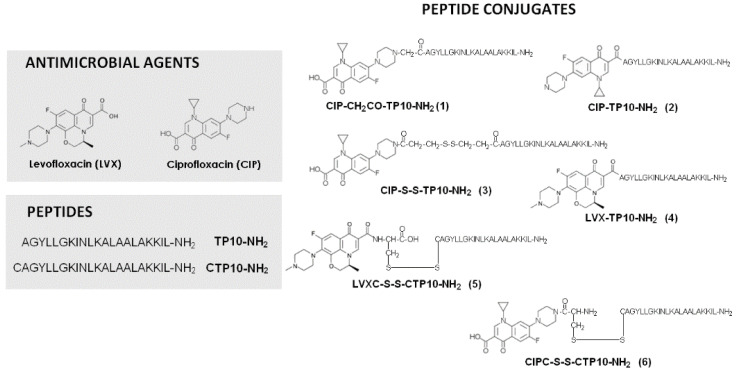
Chemical structures of the CIP and LVX conjugates with transportan 10 and their constituents [26].

All conjugates were synthesized according to the methodology described previously [26,30]. In the case of conjugates **2** and **4**, CIP and LVX were manually added to the peptidyl resin. *N*,*N*’-diisopropylcarbodiimide (DIC), *N*,*N*’-diisopropylethylamine, and LVX or Boc-CIP (3 equiv. of each) were dissolved in an equimolar amount of DMF/DCM, put in the SPPS vessel with the peptidyl resin, and mixed for 90 min. This procedure was repeated until the chloranil test gave a negative result. To synthesize conjugate **2**, the submonomeric approach [30] was utilized. In the first step, bromoacetic acid and DIC (5 equiv. of each) in DCM/DMF (1/1; *v/v*) were added to the peptidyl resin and stirred in the SPPS vessel for 30 min. This procedure was repeated twice. The coupling of ciprofloxacin to the peptidyl resin was achieved by adding a suspension of CIP (1.5 equiv.) and triethylamine (1.5 equiv.) in DCM/DMF. The coupling reaction took 24 h at room temperature. Conjugate **3** was obtained in a two-step procedure. Firstly, Lomant’s reagent (DSP) (1.2 equiv.) was dissolved in DMF, added to the SPPS vessel with the peptidyl resin, and shaken 24 h. This procedure was repeated twice. In the next step, ciprofloxacin and triethylamine (1.5 equiv. each) were dissolved in an equimolar amount of DMF/DCM and added to the peptidyl resin with an attached DSP. The coupling took another 24 h at room temperature. Finally, all the conjugates were cleaved from the resin and purified as described before. In order to obtain conjugates **5** and **6**, the disulfide bridge formation between LVX and CIP and CTP10-NH_2_ was preceded by the coupling of the antibiotic to the Cys derivative. To obtain conjugate **5**, LVX-Cys (Npys) (12 mg, 0.02 mmol) was dissolved in 5 mL of DMF, and CTP10-NH_2_ (46 mg, 0.02 mmol) was added, and the reaction was mixed for 4 h at room temperature. The progress of the reaction was examined by analytical HPLC. After 4 h, the solvent was removed in vacuo, and the conjugate was purified by semipreparative HPLC. In the case of conjugate **6**, the disulfide bridge was formed during the reaction of CTP10-NH_2_ and Cys(Npys)-CIP, as described above for **5**. In Figure 1, the structures of the CIP and LVX conjugates are presented.

### 4.2. HPLC Analysis

**Figure 2:**
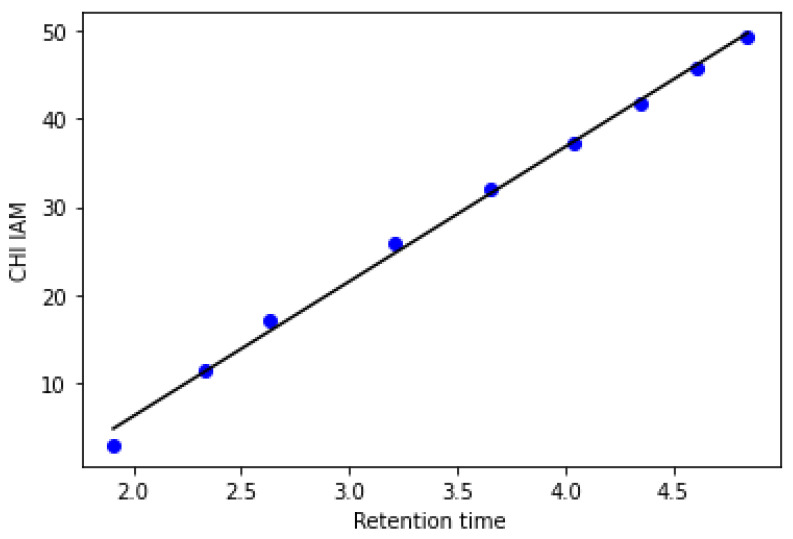
Calibration cure of the IAM-HPLC: acetaminophen (t_R_ 1.93, CHI_IAM_ 2.90), acetanilidine (t_R_ 2.37, CHI_IAM_ 11.50), acetable (t_R_ 2.66, CHI_IAM_ 17.20), propiohenone (t_R_ 3.22, CHI_IAM_ 25.90), butyrophenone (t_R_ 3.67, CHI_IAM_ 32.00), valerophenone (t_R_ 4.04, CHI_IAM_ 37.30), hexanophenone (t_R_ 4.35, CHI_IAM_ 41.80), heptanophenone (t_R_ 4.61 CHI_IAM_ 45.70), and octanophenone (t_R_ 4.83, CHI_IAM_ 49.4).

All HPLC experiments were carried out using a Prominence-1 LC-2030C 3D HPLC system (Shimadzu, Tokyo, Japan) equipped with an IAM.PC.DD2 column (10 mm × 4.6 mm; particle size 10.0 µm with an IAM guard column; Regis Technologies, Austin Ave, Morton Grove, IL, USA) and diode array detection (DAD). The HPLC system was controlled by LabSolution software (version 5.90, Shimadzu, Japan). The stock solutions of the solutes were diluted to obtain 100-µg/mL concentrations, and the injected volume was 10 µL, which was used for the analytes. The IAM-HPLC analyses were performed with a linear gradient of 0–85% in phase B (where phase A was 10-mM phosphoric buffer at pH 7.4, and phase B was acetonitrile) at a flow rate of 1.5 mL/min. The ultrapure water used for the mobile phase preparation was purified by the Millipore Direct-Q 3 UVWater Purification System (Millipore Corporation, Bedford, MA, USA). The other reagents used for the preparation of the mobile phase: acetonitrile, sodium phosphate dibasic dehydrate, and sodium phosphate monobasic monohydrate (Sigma-Aldrich, Steinheim, Germany) were analytical grade. During the chromatographic analysis, the temperature of the column was constant at 30.0 °C, and the analysis time was 6.5 min. The CH_IAM_ indices of the target solutes were obtained using a calibration set of the reference substances using the protocol developed by Valko and coworkers [13,14]. The reference substances were purchased, respectively: octanonophenone and butyrophenone acetanilide (Alfa Aesar, Haverhill, MA, USA); acetaminophen, acetophenone, levofloxacin, and ciprofloxacin (Sigma-Aldrich, Steinheim, Germany); and heptanophenone, hexanophenone, valerophenone, propiophenone, and acetophenone (Acros Organic, Pittsburg, PA, USA). Figure 2 presents a correlations plot of the CHI_IAM_ indices of the model substances and experimentally determined retention times.

## 5. Conclusions

IAM-HPLC may be a valuable tool for the characterization of antimicrobial peptide origin derivatives. Although, in the case of short cationic lipopeptides and conjugates of CIP and LVX with TP10-NH_2_, the results only have a qualitative nature, they can broaden the inference about their mechanisms of action. Nevertheless, the obtained results should be applied with care, since the surface of the IAM phase only simplifies the nature of the cell membrane. From a practical point of view, another critical observation is that a strong interaction between short cationic lipopeptides and the IAM stationary phase can be utilized to develop an effective method for their purification. Furthermore, IAM-HPLC can be used for the rapid screening of the physicochemical properties of citropin analogs. In f2the case of this class of chemical structures, it could be recommended as a screening method for the further optimization of citropin derivatives.

## Figures and Tables

**Table 1:** The obtained retention times and calculated CHI_IAM_ for the target citropin analogs and fluoroquinolone antibiotics.

Analyte	T_R1_	T_R2_	T_R3_	Mean T_R_	CHI_IAM_
(4–16) Citropin	3.987	3.979	3.985	3.984	38.24
(8–16) Citropin	4.482	4.496	4.506	4.495	45.95
(1–7) Citropin	4.240	4.244	4.244	4.243	42.15
(4–14) Citropin	3.305	3.289	3.293	3.295	27.85
(1–7)–(10–16) Citropin	3.888	3.901	3.934	3.908	37.09
(1–5)–(12–16) Citropin	3.349	3.348	3.351	3.349	28.66
Levofloxacin	3.087	3.089	3.101	3.092	24.88
Ciprofloxacin	2.886	2.921	2.899	2.902	19.70

**Table 2:** Amino acid sequences of the studied antimicrobial lipopeptides.

Double Fatty Acid Chain Lipopeptides	Tetradecanoic Fatty Acid Lipopeptides	Hexadecanoic Fatty Acid Lipopeptides
(C_8_)_2_-KKKK-NH_2_	C_14_-K-NH_2_	C_16_-K-NH_2_
(C_10_)_2_-KKKK-NH_2_	C_14_-KG-NH_2_	C_16_-KGK-NH_2_
(C_12_)_2_-KKKK-NH_2_	C_14_-KGK-NH_2_	C_16_-KGKG-NH_2_
(C_14_)_2_-KKKK-NH_2_	C_14_-KGKG-NH_2_	C_16_-KK-NH_2_
(C_16_)_2_-KKKK-NH_2_	C_14_-KKK-NH_2_	C_16_-KKKK-NH_2_
	C_14_-KKKK-NH_2_	C_16_-KKY-NH_2_
		C_16_-KKS-NH_2_
		C_16_-KKD-NH_2_

**Table 3:** Amino acid sequences of the studied analogs of citropin 1.1.

Antimicrobial Peptides	Amino Acid Sequences
Citropin 1.1	GLFDVIKKVASVIGGL-NH_2_
(4–16) Citropin	DVIKKVASVIGGL-NH_2_
(8–16) Citropin	KVASVIGGL-NH_2_
(1–7) Citropin	GLFDVIK-NH_2_
(4–14) Citropin	DVIKKVASVIG-NH_2_
(1–7)–(10–16) Citropin	GLFDVIKASVIGGL-NH_2_
(1–5)–(12–16) Citropin	GLFDVVIGGL-NH_2_

## Data Availability

All data are presented in Table 1.
